# Improved BP Neural Network Ensemble Model for Asphalt Pavement Performance Prediction

**DOI:** 10.3390/ma19153245

**Published:** 2026-07-31

**Authors:** Xinyu Zuo, Yufan Du, Guangsheng Zeng, Ahmed D. Almutairi

**Affiliations:** 1 School of Architecture and Design, China University of Mining and Technology, Xuzhou 221000, China; 18796331435@163.com; 2 College of Civil Engineering, Fuzhou University, Fuzhou 350108, China; 18359321220@163.com (Y.D.); 18065140524@163.com (G.Z.); 3 Department of Civil Engineering, College of Engineering, Qassim University, Buraydah 52571, Saudi Arabia

**Keywords:** asphalt pavement, pavement performance prediction, BP neural network, machine learning

## Abstract

With the growing complexity and variability of the operational environment of asphalt pavements and the continuous increase in traffic loads, traditional pavement performance prediction models cannot accurately depict the nonlinear degradation process of pavement performance with the passage of time. Therefore, a novel approach is proposed in this paper to forecast the service performance of asphalt pavements accurately. This method optimises a Backpropagation (BP) neural network using the Levenberg–Marquardt (LM) algorithm. Seven main influencing factors were selected as the input parameters to construct the prediction model, and the performance of the prediction model was evaluated. The parameters considered in this analysis are: road age, average daily traffic volume for one year, annual temperature range, annual precipitation, relative humidity, pavement thickness and pavement surface compressive strength. Through these factors cumulatively, the model is able to predict and evaluate the road condition and Pavement Quality Index (PQI) accurately. The results indicate that the proposed model is better than the baseline model of traditional BP neural networks in predicting the Road Condition Index (RCI), with a Mean Absolute Error (MAE) of 0.395. This is an important reference to predict the service performance of asphalt pavements and validate the effectiveness of the model.

## 1. Introduction

The issue of performance degradation of asphalt pavements is becoming increasingly severe due to the continuous increase in highway traffic loads, the complexity of climatic conditions, and the extended service life of roads. One of the major challenges in the field of highway engineering is the formulation of accurate maintenance measures based on the mechanisms underlying the evolution of pavement performance. This requires an in-depth understanding of these mechanisms. In the United States, Japan and Germany, research on the performance of asphalt pavements has shifted from the traditional experience-based design approach to data-driven methods involving intelligent prediction and big data analysis [1]. This is a big change in highway engineering, both in theory and in practice. The primary aim of initial research was to develop empirical, statistical regression and mechanistic models for quantifying and understanding the complicated relationships between pavement damage, traffic load intensity and environmental factors. This approach provided a good evaluation of the pavement performance. The Long-Term Pavement Performance (LTPP) program and the wealth of road data it has generated has stimulated research on proactive maintenance technologies and comprehensive life-cycle management strategies [2,3]. In recent years, prediction methods via fusion of multi-source data have become a popular research topic in academia since the rapid development of digital technologies, deep learning, and machine learning [4,5]. Road maintenance management has evolved from a rough and extensive approach to a more refined and precise one by introducing various information, such as traffic volume, meteorological conditions, structural characteristics and maintenance history, to establish an accurate mathematical model of the road performance degradation process [6,7].

**Figure 1:**
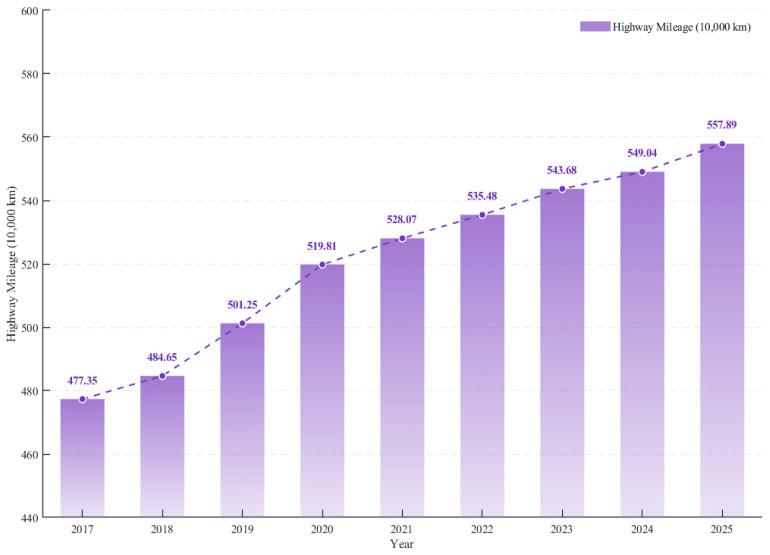
China’s highway mileage, 2017–2025.

The construction of asphalt highways in China started relatively late, but it has made rapid progress and achieved remarkable results since then. As shown in Figure 1, this paper compiles the data of China highway mileage from 2017 to 2025. As the total highway mileage has increased, the emphasis of highway construction has shifted from building new infrastructure to maintaining existing assets. Road management strategies are experiencing a major transformation, shifting from traditional experience and intuitive judgement to decision-making based on scientific prediction and data analysis. The academic research in China currently mainly focuses on pavement technical condition evaluation, performance degradation mechanism investigation, optimal maintenance scheduling, and predictive model development. The majority of the studies apply advanced methodologies, such as artificial neural network architectures, time-series statistical analysis, fuzzy systems theory and ensemble learning techniques [8,9]. However, the existing methods still have some challenges, like being sensitive to random disturbance, having difficulty in accurately characterising the nonlinear relationships and poor stability in long-term prediction because of the complex mechanism of pavement degradation, the strong interaction among variables and the variability of monitoring data [10].

Traditional methods of forecasting asphalt pavement performance include empirical statistical models, regression analysis models, time series analysis models, fuzzy prediction models and mechanistic models. But the most conspicuous demerits of conventional methods are as follows:

(1) Empirical statistical methods are based on fitting with historical data. They have the advantages of fast calculation and easy use, but they cannot accurately describe complex nonlinear degradation mechanisms [11,12,13].
(2) Regression analysis models are used to establish relationships between performance indicators and influencing factors, but they have poor generalisation ability and impose too strict assumptions about the independence and linearity of variables [14,15].
(3) Road performance trends over time can be described using time series analysis techniques. In general, this method is concerned with internal time dependencies and does not take into account the effect of many external factors, such as traffic volume and environmental conditions [16,17].
(4) Fuzzy models are suitable for prediction tasks with small sample sizes; they are sensitive to random disturbances and often have no flexibility and effectiveness when interacting with complex systems [18,19].

Therefore, it is necessary to develop an asphalt pavement performance prediction model to effectively balance engineering applications, model robustness and prediction accuracy. The advantages of the BP neural network prediction model optimised for LM in this paper are as follows:

(1) Faster convergence of models: The LM algorithm is able to search the parameters quickly by dynamically modifying the damping coefficient, based on the ideas of optimisation based on the Gauss–Newton method and gradient descent. This greatly reduces the training time and also increases the computational efficiency of the model.
(2) Improved prediction accuracy: The LM optimisation algorithm is more successful in finding the minimum of the error function, thus further enhancing the capability of the model to represent complex nonlinear relations and improving the fitting capability of the network parameters. It reduces the prediction errors effectively.
(3) Enhanced robustness and stability of the model: Traditional BP neural networks are unstable during training due to initial weights and sample fluctuations, which significantly reduce the learning speed and effectiveness of traditional BP neural networks. The LM algorithm can considerably enhance the model’s overall robustness and reliability, as well as the stability of weight updates, by implementing an approximately second-order optimisation process. This avoids the situation where local outliers and random noise cause large deviations in the model prediction results.

The purpose of this paper is to identify the factors that influence the service performance of asphalt pavements by collecting and analysing data on asphalt pavements, climate conditions, and geographical locations. In accordance with the aforementioned, BP neural network technical means are chosen to construct an analysis system that is primarily employed to accurately predict and evaluate the service performance of asphalt pavement under a specific climate. Then, the LM algorithm is used to perform optimised training to improve the model’s predictive accuracy and generalisability. To speed up the completion of maintenance and repair work and ensure that the service quality of asphalt pavements is at a high level, it is necessary to carry out comprehensive evaluations and inspections on road sections where pavement performance has been identified [20,21,22]. To achieve sustainable road infrastructure management, it is important to minimise the arbitrariness of asphalt pavement maintenance operations, to use accurate measures to optimise resource utilisation, and to ensure that maintenance activities are consistent with scientific principles [23,24]. Due to the inherent complexity and uncertainty of the bitumen performance prediction, a strengthened BP neural network performance prediction model is proposed. This model has the characteristics of self-organization and self-learning and has strong versatility, fast solution speed and good fault tolerance.

## 2. Related Work

Meieret et al. [25] applied neural network technology to the process of inverting road surface properties for the first time, resulting in a significant increase in the speed of this inversion. This method is now much faster than previous traditional methods, with a speedup of approximately 1500 to 2200 times. Subsequently, Meier et al. subsequently increased the performance of surface layer elastic modulus inversion by a factor of 42 by embedding a fully trained neural network model into the WESDEF inversion system. Tang et al. [26] proposed a monthly decay characteristic GM(1, 1) Grey Model that combines the Pavement Condition Index (PCI) and the Performance Quality Index (PQI) in order to enhance the model’s performance in complex environments. This approach was derived from the observation that the GM(1, 1) Grey Model and machine learning algorithms are susceptible to computational errors under high condition number conditions. It is evident that this method can effectively reduce the condition number of the matrix while ensuring relatively small errors, as evidenced by the results of tests conducted using historical highway data from Hunan Province. Khawaga et al. [27]. validated two distinct prediction models: the Sigmoid method, which is based on the S-curve model, and the Markov chain method, which is based on probability theory, by utilising actual IRI measurement data from 44 flexible pavement sections in the GPS-1 project within the LTPP database. The study suggests that the two methods show very similar performance in terms of the prediction accuracy, although they differ in their specific implementation. Wen et al. [28]. studied the mechanisms that lead to the reduction of asphalt pavement performance after maintenance in depth. They developed a prediction model by which they could accurately predict pavement performance trends using auto-regressive integrated moving average (ARIMA) time series analysis. The model enhanced the scientific rigour and effectiveness of the maintenance decision making to a great extent. The model maintained the initial prediction error within 5% and achieved accurate predictions at a 95% confidence level. Guo et al. [29]. proposed a Gradient Boosting Decision Tree (GBDT) prediction model which can predict the rut depth under the effect of various influence factors. Chuan et al. [30]. Models trained using data from the U.S. Department of Transportation’s LTPP database were used to compare the predictive accuracy of an encoder-decoder architecture based on Gated Recurrent Unit (GRU) network layers, the classic XGBoost machine learning model, and a single Long Short-Term Memory (LSTM) network. The results showed that the architecture based on the GRU had better prediction performance. Lu et al. [31]. showed that the BP neural network has a strong ability to predict the rebound modulus of different layers of road surfaces accurately with certain input and output parameters. This greatly improves the scientific rigour and effectiveness of road maintenance and management. Ethan et al. [32] improved the accuracy and training speed of a BP neural network by increasing its learning rate using genetic algorithms.

## 3. Methodology Architecture

**Figure 2:**
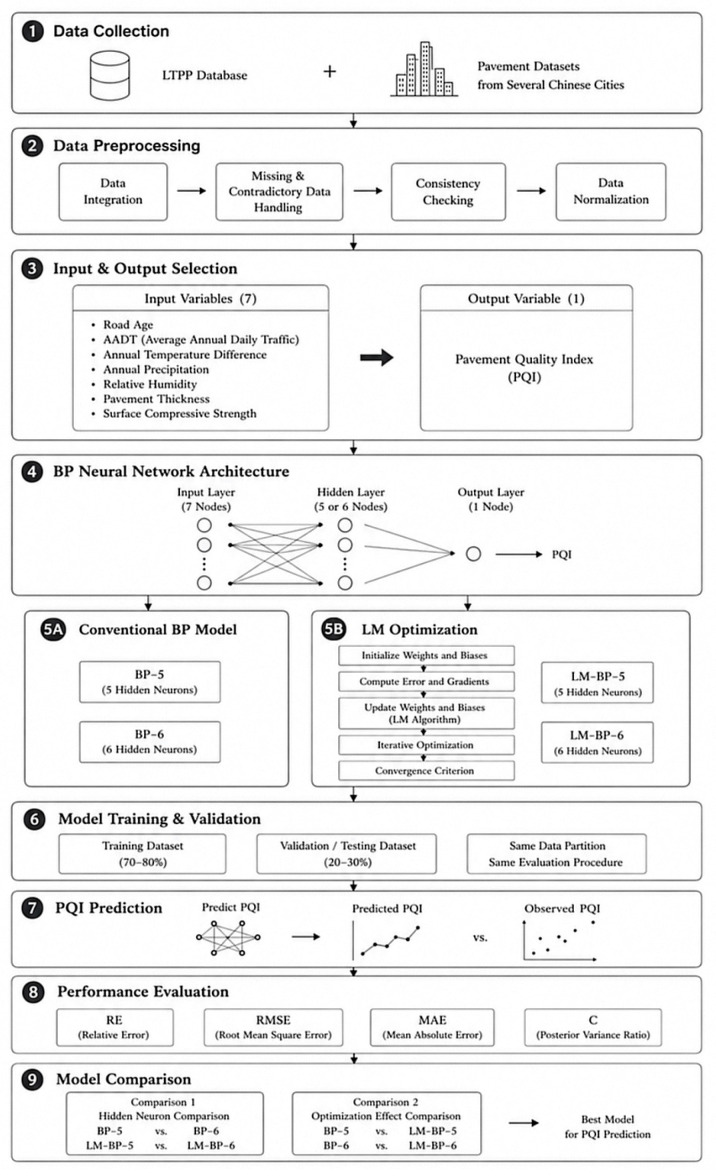
Overall workflow.

Figure 2 shows the suggested comprehensive methodological approach of this research. The procedure includes eight major steps: (1) data collection, (2) data preprocessing, (3) selection of input and output variables, (4) construction of the BP neural network, (5(A,B)) optimization of the BP neural network by the Levenberg–Marquardt (LM) algorithm, (6) model training and validation, (7) prediction of pavement performance, and (8) evaluation and comparison of model performance.

First, the data of asphalt pavement performance were collected from the Long-Term Pavement Performance (LTPP) database and pavement datasets of some cities in China. The collected data consisted of information on pavement structural characteristics, material properties, traffic conditions, environmental conditions and pavement performance. The datasets were subsequently combined into one database. In the data preprocessing, the missing and inconsistent data was identified and removed or treated appropriately, and the data was checked for consistency and reliability. The curated dataset was then structured for future model development.

Secondly, the selection of the input and output variables for the predictive model was based on the availability of the data and their expected engineering significance to the efficiency of asphalt pavements. The input variables (i.e., seven quantitative variables) selected included road age, Average Annual Daily Traffic (AADT), annual temperature variation, annual precipitation, relative humidity, pavement thickness and surface compressive strength. The asphalt pavement’s overall service performance was represented by the Pavement Quality Index (PQI), which was chosen as the output variable. Then, a BP neural network was used to reflect the correlation between these impacting elements and PQI.

Third, a BP neural network was built using the seven selected input variables, with PQI as the outcome variable. The network designs with different dimensions of the hidden layer were compared, and the ones with five and six hidden neurons were selected for comparative study. The weights and biases of the classical BP neural network were updated iteratively so as to minimize the prediction errors.

Fourth, the Levenberg–Marquardt (LM) method was introduced to improve the optimization efficiency and predictive ability of the traditional BP neural network. The weights and biases were updated by the LM algorithm in the iterative training process to improve the convergence properties of the network. The LM-optimized BP models with five and six hidden neurons were built under the same experimental conditions as the traditional BP models.

Fifth, the assembled pavement dataset was used to train and validate the BP and LM-optimized BP models. In order to ensure a fair comparison, the same data segmentation and evaluation methods were used across several model setups. The developed models were then used to predict the corresponding asphalt pavement section’s PQI values.

Finally, the predicted PQI values were compared to the real values in order to evaluate the accuracy of the predictions and the performance of the model. The models were assessed on the basis of the metrics of Relative Error (RE), Root Mean Square Error (RMSE), Mean Absolute Error (MAE) and the posterior variance ratio (C). Comparative tests were used to examine the impact of hidden-layer dimensions and the role of the LM optimization algorithm on the predictive efficacy of the BP neural network.

The whole procedure is illustrated in Figure 2. It included data collection and preprocessing, variable selection, BP neural network development, LM optimization, model training and validation, PQI forecasting and performance assessment.

## 4. Improved Model

### 4.1. BP Neural Network

**Figure 3:**
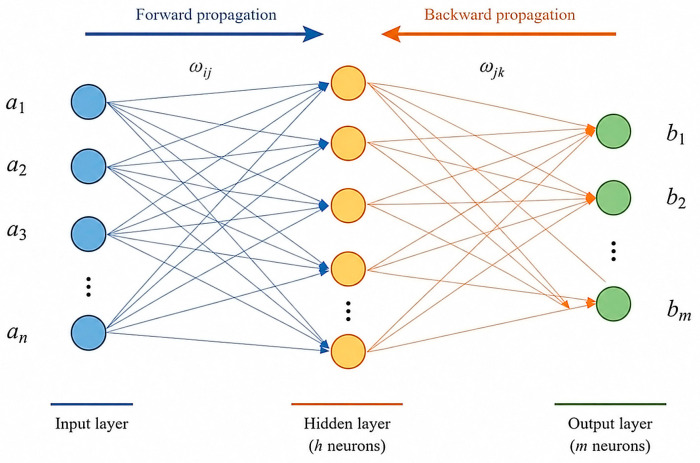
Diagram of a BP neural network architecture.

The BP neural network is a commonly used nonlinear modelling approach that can approximate the complicated nonlinear relations between input variables and output responses. During the training phase, the network continuously adjusts the weights of the connections and biases in order to learn the relationship between inputs and outputs [33,34,35]. The BP neural network has an input layer, one or more hidden layers and an output layer, as shown in Figure 3. In the forward propagation phase, the input variables go through the network step by step. The errors between expected and actual outputs are fed back to change the network parameters.

**Figure 4:**
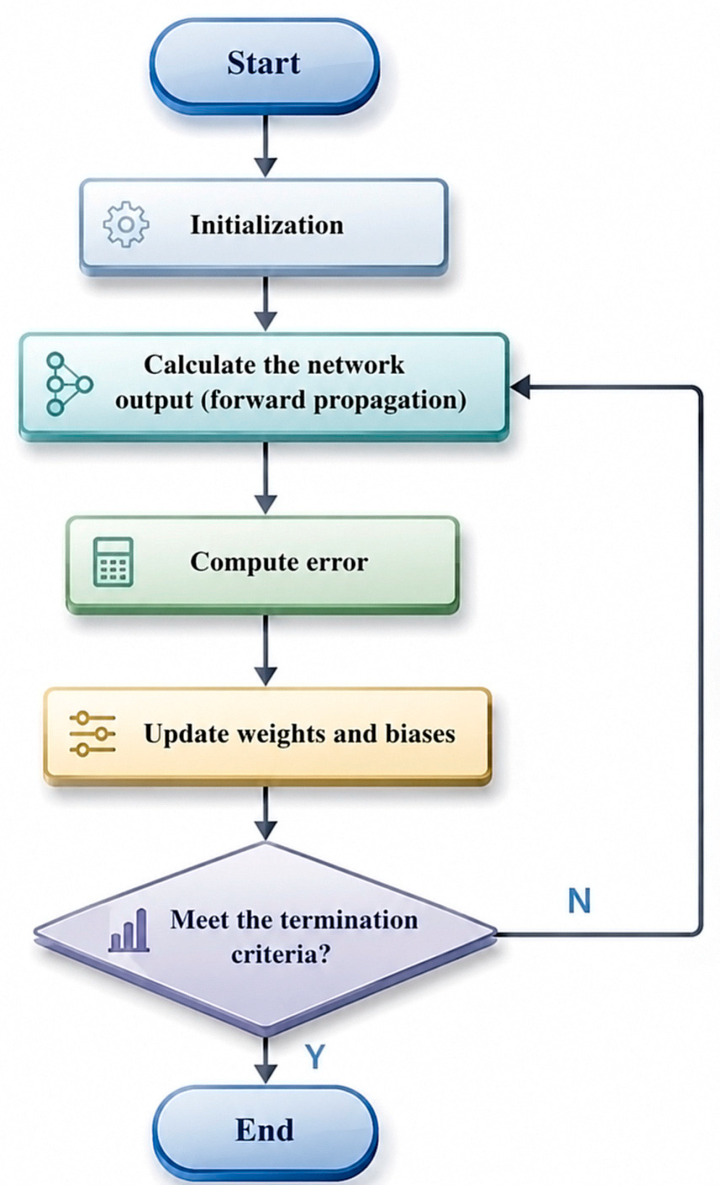
BP neural network flowchart.

In this paper, the nonlinear relationship between the determinants of asphalt pavement performance and PQI is described by the BP neural network. The seven input parameters were road age, average annual daily traffic (AADT), yearly temperature fluctuations, annual rainfall, relative humidity, and pavement thickness and surface compressive strength. The PQI was selected as the sole output variable to reflect the entire service performance of asphalt pavement. The interaction between traffic load, ambient conditions, pavement structural attributes and aging mechanisms could lead to a nonlinear relationship between these input factors and PQI. Therefore, the BP neural network is suitable for the predictive problem discussed in this paper because of its nonlinear approximation ability. The BP neural network is usually trained by a gradient descent-driven error backpropagation algorithm. The basic training procedure is illustrated in Figure 4 and can be summarized as shown.

Building a Neural Network Model: 

(1) Initialization. Given weights *wij* (*p*), biases *bi* (*p*), learning rate *η*, and activation function *f*, where *z_i_* (*p*) represents the weighted input, the neurons in the *p* layer are defined by Equations (1) and (2):

(1) $$z_{i}^{\left(p\right)}=\sum_{j}\omega_{ij}^{\left(p\right)}a_{i}^{\left(p-1\right)}+b_{i}^{\left(p\right)}$$

(2) $$a_{i}^{\left(p\right)}=f\left(z_{i}^{\left(p\right)}\right)$$

(2) Calculation of Output Error. Suppose the true output is *yk*, the actual output is *ak* (*p*), and the error term εi (*p*) is given by Equation (3):

(3) $$\varepsilon_{i}^{\left(p\right)}=\sum_{j}\omega_{ji}^{\left(p+1\right)}\varepsilon_{j}^{\left(p+1\right)}\cdot f^{\prime }\left(z_{i}^{\left(p\right)}\right)$$

(3) Weight adjustment. The weights *w*′*ij* (*p*) and biases *b*′ *i* (*p*) are adjusted according to Equations (4) and (5):

(4) $${\omega^{\prime }}_{ij}^{\left(p\right)}=\omega_{ij}^{\left(p\right)}-\eta \varepsilon_{i}^{\left(p+1\right)}\cdot a_{j}^{\left(p\right)}$$

(5) $${b_{i}^{\prime }}_{i}^{\left(p\right)}=b_{i}^{\left(p\right)}-\eta \varepsilon_{i}^{\left(p+1\right)}$$

(4) Training ends when the error falls below a specified value or the specified number of training iterations is reached; otherwise, repeat Equation (1).

The training procedure ends when the error limit is reached or the maximum number of iterations is reached. Otherwise, the forward propagation and error backpropagation steps are iterated.

Traditional BP neural networks possess strong nonlinear approximation capabilities, but the gradient descent training methodology has many limitations. The convergence might be slow at first, especially if the error landscape is complex or the starting parameters are not optimal. Secondly, the optimization process may be trapped in a suboptimal solution, limiting the stability and prediction accuracy of the trained. Thirdly, the performance of BP networks depends on the choice of training parameters and the design of network architecture. These limitations are especially important for the prediction of asphalt pavement performance because the relationships between pavement age, traffic loading, climate conditions and structural properties are nonlinear and possibly interrelated. Therefore, it is necessary to optimize the BP neural network so as to improve the parameter adjustment and convergence characteristics of it. The number of neurons in the hidden layer is a key hyperparameter which affects the learning ability, model complexity, prediction accuracy and generalization ability of a BP neural network. A small number of neurons in the hidden layer may not allow the network to model complex nonlinear relationships, while a large number may increase the computational costs and the risk of overfitting. An appropriate size of hidden layer should trade off the nonlinear representation ability, model complexity and computational efficiency.

The research involved a systematic comparison of two candidate topologies of hidden layer with five and six neurons, respectively. Instead of assuming that there were mathematically ideal architectures, these combinations were considered as potential architectures. The two network architectures were trained and tested using the same data partition and modelling parameters. Such a configuration allowed for an analysis of the effect of hidden-layer dimensions on the predictive efficacy of the BP neural network while ensuring a uniform network design for further comparison with the LM-optimized BP models.

If you have too many nodes in the hidden layer, you can have overfitting and a longer training time. *K* is the best number of hidden layer nodes, *i* is the number of input layer nodes and *j* is the number of output layer nodes. 

**Figure 5:**
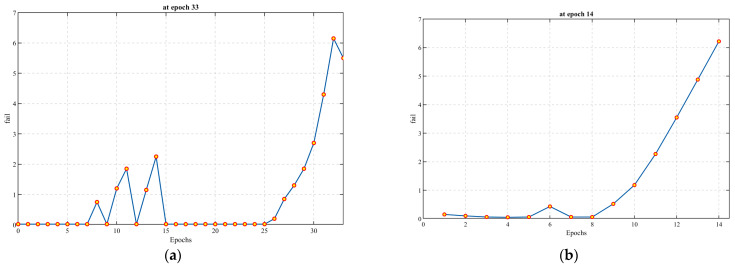
Training diagrams for BP neural networks with 5 (**a**) and 6 (**b**) hidden nodes.

The output layer, *j*, contains one node, while the input layer, *i*, contains seven. The research was a systematic study of two prospective hidden layer topologies with five and six neurons, respectively. Consequently, the BP neural network model was trained and modelled with hidden layer node counts of five and six, respectively, as illustrated in Figure 5.

### 4.2. LM Algorithm Optimization

The traditional BP neural network is basically a parameter update based on gradient descent. Although this approach is quite simple to implement, it might need many iterations to converge and may show erratic optimization patterns for complicated nonlinear features in the error landscape. Such constraints can affect the effectiveness of training as well as the prediction accuracy.

To overcome these limitations, the Levenberg–Marquardt (LM) algorithm was designed to optimize the weights and biases of the BP neural network. The LM approach has been chosen because the pavement performance prediction problem addressed in this research has a nonlinear regression nature with a continuous PQI output and can be formulated as a nonlinear least-squares optimization problem. LM algorithm has both gradient descent and Gauss–Newton optimization properties. Hence, it can provide more stable parameter updates compared to the traditional gradient descent, and faster local convergence when the optimization is close to a good solution.

Unlike the classical BP algorithm, LM uses the local curvature information in the Jacobian matrix of the residual vector to find the direction of parameter updates. This allows the algorithm to make more informed changes to the weights and biases of the network. Furthermore, the damping parameter enables the Levenberg–Marquardt algorithm to switch between the steepest descent and the Gauss–Newton method depending on the local shape of the error landscape. Increasing the damping parameter makes the update more conservative, similar to gradient descent. Decreasing the damping parameter allows the algorithm to move towards the faster Gauss–Newton optimization framework. This adaptive system enhances the stability and convergence properties of the optimization process.

The computing capacity of the proposed network also influences the LM choice. The network used in this research is quite small. It has seven input neurons, five or six hidden neurons and one output neuron. While the Jacobian matrix and matrix inversion in LM still require more calculations, the computational load is manageable for a network of this size. Hence, the potential enhancement of convergence efficiency and prediction accuracy presents a reasonable trade-off between computational cost and model performance for the pavement prediction problem discussed in this study.

(1) Given the weight matrix *w*, the bias vector *b*, and the regularization parameter *δ*, calculate the outputs of the hidden layer and the output layer, according to Equation (6):

(6) $${\hat{y}}_{i}=f\left(Wx_{i}+b\right)$$

(2) The gradients of the weight matrix and the bias vector are computed by Equations (7) and (8):

(7) $$\nabla_{W}E=\frac{1}{q}\sum_{i=1}^{q}\left(y_{i}-{\hat{y}}_{i}\right)\cdot x_{i}^{T}\cdot f^{\prime }\left(Wx_{i}+b\right)$$

(8) $$\nabla_{b}E=\frac{1}{q}\sum_{i=1}^{q}\left(y_{i}-\hat{y_{i}}\right)\cdot f^{\prime }\left(Wx_{i}+b\right)$$

(3) The inverse of the Hessian matrix *Hk* −1 is calculated and the parameters are adjusted by Equations (9)–(12)

(9) $$\Delta W=-{\left(H_{k}+\delta I\right)}^{-1}\cdot \nabla_{W}E$$

(10) $$\Delta b=-{\left(H_{k}+\delta I\right)}^{-1}\cdot \nabla_{b}E$$

(11) $$W^{\prime }=W-\alpha \Delta W$$

(12) $$b^{\prime }=b-\alpha \Delta b$$

(4) The training process should be terminated when the error falls below a predetermined threshold, the specified number of training iterations is completed, or the gradient approaches zero. Otherwise, the iteration should proceed in accordance with Equation (1). The LM algorithm was employed to develop and optimise BP neural network prediction models in this study. The number of hidden layer nodes *k* was set to five and six, respectively. BP neural networks were constructed with the same architecture of the two hidden layers with five and six hidden neurons, and they were optimized by LM. This controlled experimental setting enabled testing of the effect of the LM optimization algorithm for different hidden layer dimensions.

Training is terminated when the prediction error falls below a predefined convergence criterion, the maximum number of iterations is reached, or the gradient is lower than a suitable level. Alternatively, the parameters are iteratively altered until the stopping criterion is met.

In order to study the separate effects of the optimization approach and the hidden-layer dimensions, two identical candidate network topologies with five and six hidden neurons were refined with the LM algorithm. Four model configurations were analysed with the same input variables, data splitting and experimental parameters, i.e., a standard BP model with five hidden neurons (BP-5), a standard BP model with six hidden neurons (BP-6), an LM-optimized BP model with five hidden neurons (LM-BP-5), and an LM-optimized BP model with six hidden neurons (LM-BP-6).

This controlled experiment enabled us to measure the effect of the LM optimization strategy in isolation for different hidden layer setups. We examined five- and six-neuron architectures as candidate configurations, choosing the final model configuration based on its experimental predictive efficiency rather than presuming it to be appropriate in advance. The proposed LM-optimized BP model was finally configured with six neurons because it performed better in prediction than the other three configurations that were tested. As per Section 6.1, the LM-BP model with six hidden neurons provided the fewest prediction errors and the best overall evaluation metrics, including the coefficient of determination (R2), Root Mean Square Error (RMSE), Mean Absolute Error (MAE), and maximum relative error. The maximum relative error was reduced to 0.71%. The R2, RMSE and MAE reached 0.991, 0.410 and 0.395, respectively. The results indicate that six hidden neurons achieved a better compromise between the nonlinear representation capability and model complexity in the dataset and experimental conditions of this work than five neurons.

The six-neuron configuration is regarded as the most effective architecture among the configurations evaluated in this research, not an ideal hidden-layer dimension in general. The accuracy of prediction is not necessarily improved by the number of hidden neuron, which may increase the computation cost and the possibility of overfitting. Therefore, when the proposed framework is applied to pavement datasets with a wide range of sample sizes, variable distributions or engineering features, the hidden layer configuration needs to be adjusted and re-evaluated.

**Figure 6:**
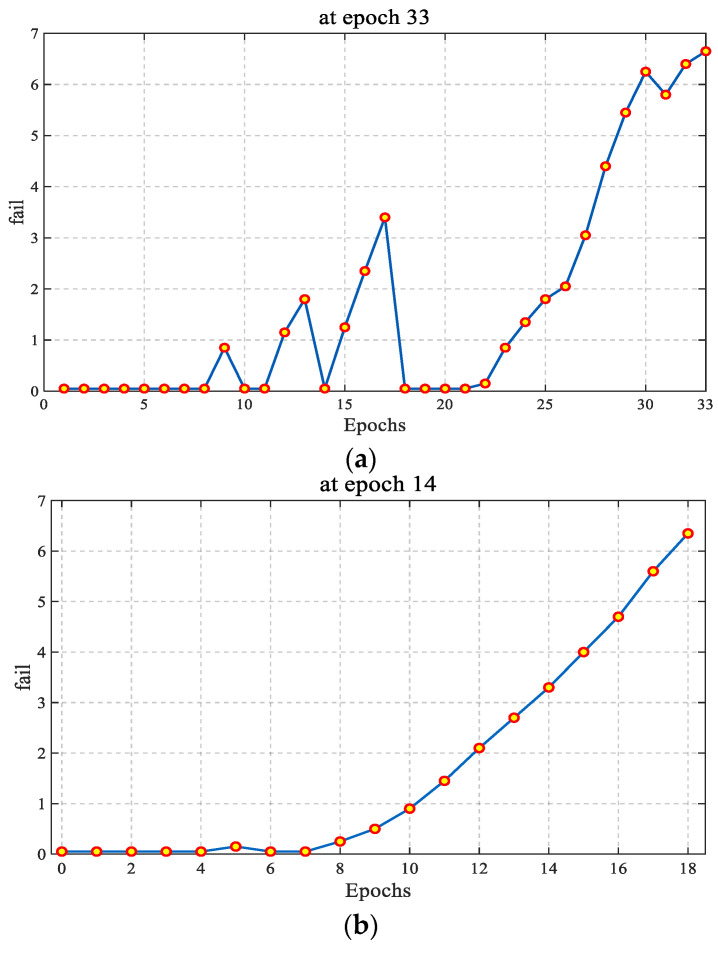
Training diagrams for BP neural networks optimized using LM with 5 (**a**) and 6 (**b**) hidden nodes.

The overall predictive performance was achieved by the LM-optimized model with six hidden neurons, as shown in Figure 6, with lower prediction errors and better evaluation metrics than all other configurations. So, the configuration with six hidden neurons was chosen as the best configuration for the final LM-optimized BP model.

### 4.3. Precision Determination Index of BP Neural Network

This study evaluated the predictive accuracy of the Long Short-Term Memory (LSTM) algorithm and Backpropagation (BP) neural network before and after optimisation using Relative Error (RE), Root Mean Square Error (RMSE) and Mean Absolute Error (MAE). These measures are used to quantify the amount of bias in the prediction at different levels of the model. The results show that the BP neural network model is more accurate in prediction work and performs better, as shown by the decreases in the RE, RMSE and MAE values. The formulas are presented in Equations (13)–(15):

(13) $$RE=\frac{\left|A_{i}-B_{i}\right|}{A_{i}}\times 100\%$$

(14) $$RMSE=\sqrt{\frac{1}{n}\sum_{i=1}^{n}{\left(A_{i}-B_{i}\right)}^{2}}$$

(15) $$MAE=\frac{1}{n}\sum_{i=1}^{n}\left|A_{i}-B_{i}\right|$$

## 5. Experimental Setup and Materials

### 5.1. Database Preparation

The asphalt pavement performance data used in this study were obtained from the Long-Term Pavement Performance (LTPP) database and additional pavement data collected from several cities of China [36]. The original datasets contain information on pavement structure, material properties, environmental conditions, traffic characteristics, construction records, maintenance activities, and pavement performance. The dataset includes road identification number, road length, structural layer thickness, material properties, pavement construction date, maintenance record, AADT and pavement performance indices, such as the Pavement Condition Index (PCI), Ride Quality Index (RQI) and Pavement Damage Index (PDI).

For the prediction model developed in this study, the input variables were selected based on data availability, completeness, consistency, and their potential engineering relevance to asphalt pavement performance. The final input variables were: road age, AADT, annual temperature difference, annual precipitation, relative humidity, pavement thickness and surface compressive strength. Road age was determined as the construction date of the pavement and the date of that pavement performance evaluation. The climatic variables were collected from the available meteorological records corresponding to the relevant pavement sections and evaluation periods. Pavement structural and material properties were characterized by surface compressive strength and pavement thickness, respectively.

It is worth mentioning that road identification numbers, construction quality records and maintenance techniques were retained as auxiliary information in the original databases, but not used as predictor variables in the final BP neural network model. Road identification numbers were removed because they are only identification information and do not represent physical or engineering characteristics of pavement performance. The last input set was also discarded (construction quality and maintenance records) due to insufficient consistency in the definitions, measurement procedures and numerical comparability across different data sources. This was done to prevent the insertion of subjective or non-comparable variables into the prediction model.

The acquired datasets were then screened, classified and combined into a single dataset. Records with missing, inconsistent or contradictory information in the selected input and output variables were deleted or processed in an appropriate manner. The dataset thus obtained was then processed for data preprocessing and normalization and then model training. The final processed dataset was utilized for the development and evaluation of the asphalt pavement performance prediction model.

Table 1 summarizes the final input and output variables of the BP neural network model according to data availability, integrity, consistency, and engineering significance.

### 5.2. Performance and Influencing Factors of Asphalt Highway Pavement

Table 2 shows that the BP neural network model has seven input variables, including road age, AADT, annual temperature variation, annual precipitation, relative humidity, pavement thickness, and surface compressive strength. The model predicts only the Pavement Quality Index (PQI) as the output variable. The formula is presented in Equation (16):

(16) PQI=f(Age,AADT,ΔT,P,RH,H,CS)

The interactions between traffic loads, structural characteristics, material properties, pavement deterioration and environmental factors affect the functional performance of asphalt pavements [37,38]. In this research, the input variables for the BP neural network model were determined by seven factors, including road age, AADT, annual temperature variation, annual precipitation, relative humidity, pavement thickness and surface compressive strength. The output variable, Pavement Quality Index (PQI), was selected to represent the overall service performance of the asphalt pavement.

The relationship of model inputs to pavement effectiveness can be written as (PQI is a function of Age, AADT, ΔT, P, RH, H and CS). (Age) is the age of the road, (AADT) is the average annual daily traffic, (ΔT) is the annual temperature variation, (P) is the yearly rainfall, (RH) is the relative humidity, (H) is the pavement thickness, and (CS) is the surface compressive strength. The nonlinear mapping relationship between the seven input variables and their corresponding PQI values was established using BP neural network.

Road age was included to separate the combined effects of pavement deterioration and extended environmental exposure. AADT is the amount of traffic loading the pavement receives and is indicative of the potential for repeated vehicle loads to cause pavement deterioration. The climatic and moisture factors affecting pavement performance included annual temperature variation, annual precipitation, and relative humidity. Pavement thickness and surface compressive strength were used to demonstrate the structural and material properties of the pavement, respectively [39,40]. These factors may interact with one another and together influence the process of asphalt surface deterioration.

This paper explicitly set the model input as a vector of seven variables, and the output as a single variable. The input vector can be written as X = [Age, AADT, ΔT, P, RH, H, CS], where the output is: (Y = PQI).

Before inputting into BP neural network, the selected input variables were preprocessed and normalized. The next step was to train the model to learn the nonlinear relationship between the selected influencing variables and pavement performance. The predicted PQI values were compared with the measured PQI values to evaluate the prediction accuracy.

#### 5.2.1. Road Surface Use Performance Indicators

The Pavement Quality Index (PQI) was selected as the output variable of the BP neural network model to describe the overall service performance of asphalt pavements. Table 3 presents the RQI, PCI and PQI metrics obtained for the selected pavement sections, and Figure 6 shows their fluctuation patterns. The RQI and PCI were also kept as extra pavement performance indicators to characterize the state of the selected pavement segments, while the PQI was used as the target variable for model development and prediction.

**Figure 7:**
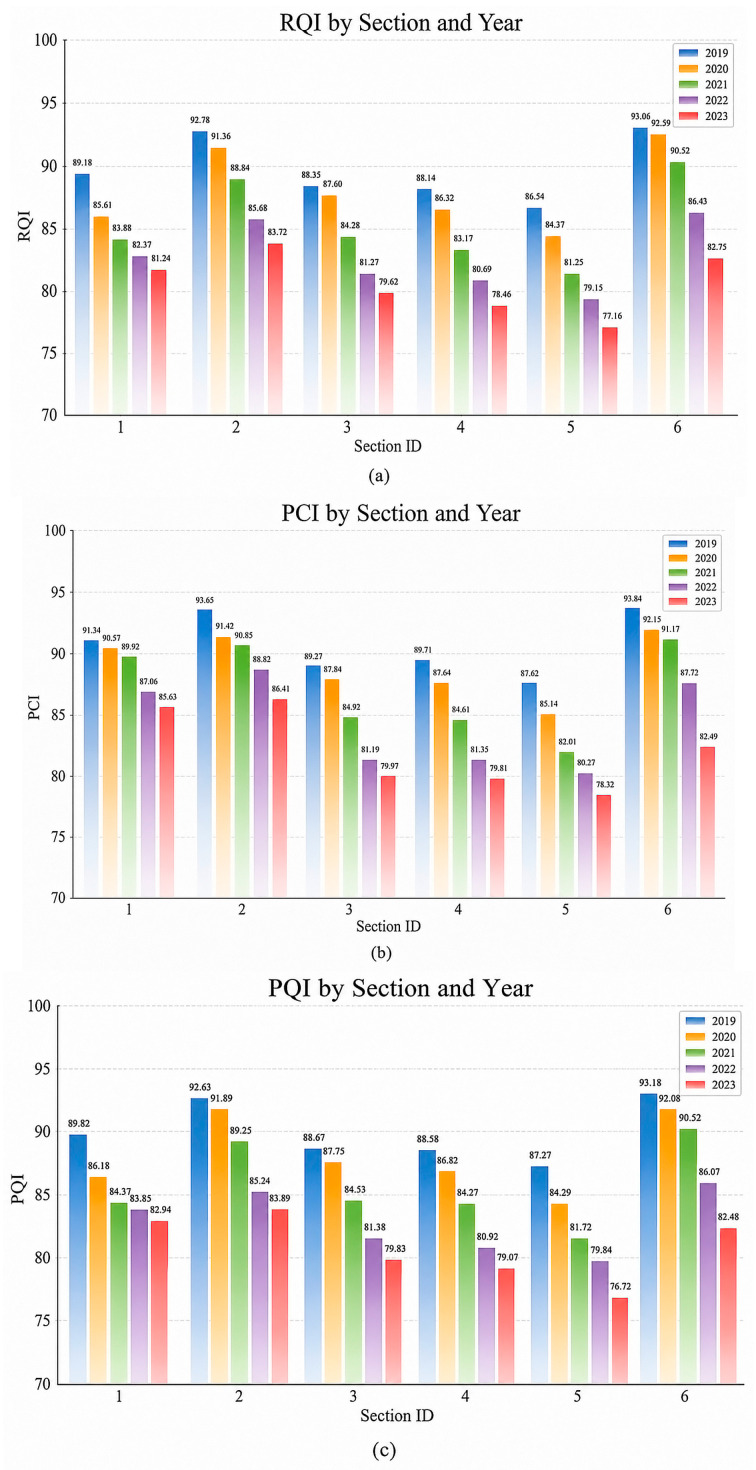
Trends in road surface RQI (**a**), PCI (**b**), and PQI (**c**).

The results in Table 3 and Figure 7 indicate that the performance of the pavement generally decreases with the age of the pavement and the cumulative traffic load. When the pavement condition is at a critical state, the degradation process may be more obvious and a sharp decline in service efficiency may occur. These findings support the use of multiple influencing factors for characterization of asphalt surface nonlinear degradation patterns.

#### 5.2.2. Traffic Volume Considerations

Traffic load is an important parameter affecting the deterioration of asphalt pavement. In this study, the AADT was selected as the quantitative indicator of traffic load and used as an input variable of the BP neural network model. In general, higher traffic volumes imply higher cumulative loading cycles and may speed up the deterioration of the pavement, particularly if the traffic is heavy for a long time.

**Figure 8:**
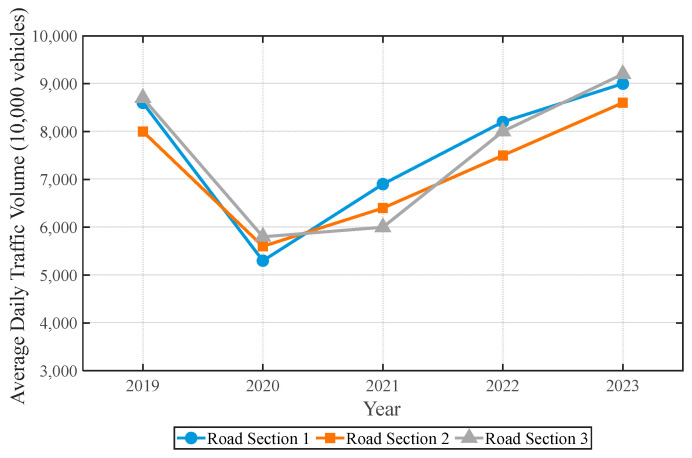
Annual average traffic volume on selected roads.

Figure 8 presents the AADT statistics for the selected pavement segments. The model examined road age and AADT simultaneously to distinguish the effect of pavement service life from that of total traffic load on pavement performance.

#### 5.2.3. Climate Factors

The degradation of asphalt surfaces is influenced by important factors, such as meteorological conditions. Three climate-related factors were taken as the input variables of the BP neural network model in this study, including annual temperature fluctuation, annual precipitation and relative humidity.

Temperature changes can affect the heat properties of asphaltic materials. Asphalt mixtures at higher temperatures soften and are more susceptible to permanent deformation and rutting, whereas at lower temperatures, the material becomes more brittle and prone to thermal cracking. Moisture-related environmental variables that could cause moisture damage and accelerate pavement deterioration were described by measuring precipitation and relative humidity.

**Figure 9:**
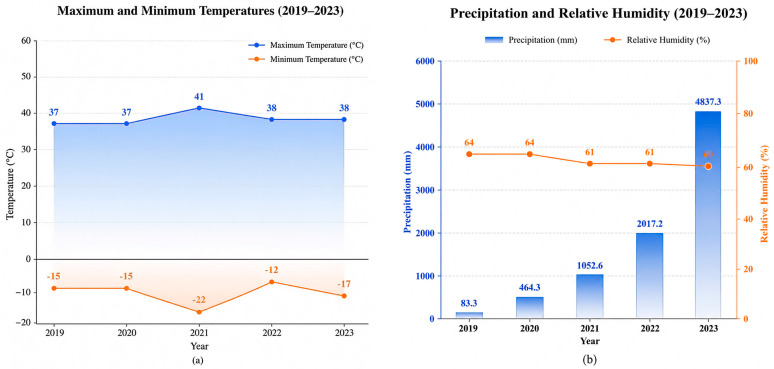
Statistics on maximum and minimum temperatures (**a**), precipitation, and relative humidity (**b**).

For each pavement segment, meteorological data were collected for the historical period and the corresponding evaluation period. Annual temperature variation was calculated as the difference between the maximum and minimum temperatures in a year, while annual rainfall and relative humidity were obtained from the meteorological data. The three variables were included in the model as numerical input parameters in a quantitative manner. The statistical distribution of peak and lowest temperature, rainfall and relative humidity for the selected pavement segments is presented in Figure 9.

#### 5.2.4. Highway Factors

The structural and material properties of pavements are the key factors affecting the load-bearing capacity and durability of asphalt surfaces. In this study, two input parameters, pavement thickness and surface compressive strength, were selected to represent the structural and material properties of the pavement.

Pavement thickness has an influence on the stress distribution and load capacity of the pavement system. A well-engineered pavement system of adequate thickness relieves the stress and deformation due to repeated traffic loads. The thickness of pavement was taken as a quantitative input variable in the BP neural network model. The details of the thickness of the pavement of the selected road segments are presented in Table 4.

The surface compressive strength was used to represent the mechanical performance of the surface layer of asphalt pavement. Generally, higher compressive strength indicates better resistance to deformation under the vehicular loads. Data on surface compressive strength were collected from typical pavement sections and entered into the model as a quantitative input variable. Table 5 summarizes the relevant findings.

## 6. Results and Discussion

### 6.1. Results and Analytical Discussion

**Figure 10:**
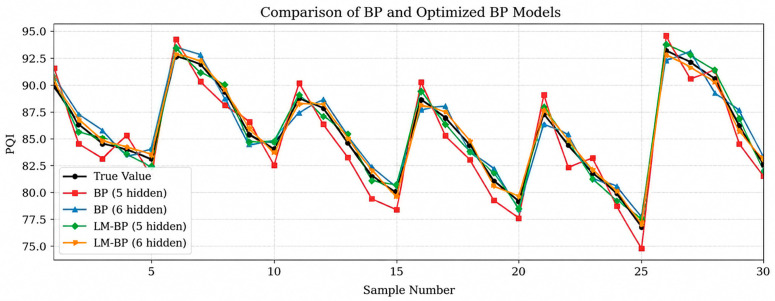
Before-and-after comparison of the optimization.

The actual and predicted values of the PQI from the classical BP and the LM-optimized BP neural networks with five and six hidden neurons are presented in Figure 10 and Table 6, respectively. The results show that LM tuning can reliably improve the prediction performance of BP neural network for all hidden layer configurations. In the five-neuron case, the maximum relative error for the conventional BP model was 2.66%, which was reduced to 0.98% for the LM modified model. With six hidden neurons, the maximum relative error decreased from 1.77% to 0.71%. Among the four tested configurations, the LM-BP model with six hidden neurons showed the best overall predictive performance, with a R2 value of 0.991, RMSE of 0.410 and MAE of 0.395.

**Figure 11:**
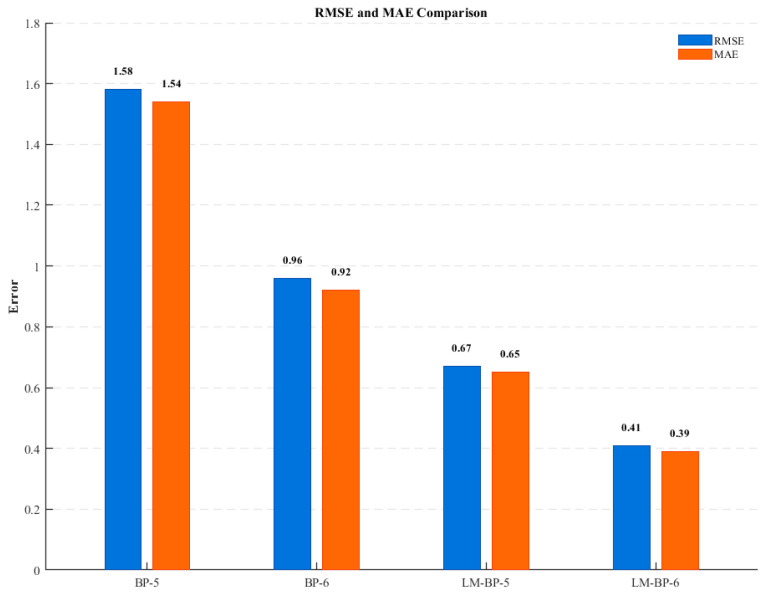
Comparison of RMSE and MAE before and after optimization.

Figure 11 also compares the RMSE and MAE of the four model settings. Simultaneous reductions in both RMSE and MAE after LM optimization prove that the improvement is not limited to one evaluation metric. The minimum prediction errors among the models considered were achieved by the LM-BP model with six hidden neurons. This indicates a better correlation between the expected and the real PQI values.

The LM-BP model’s higher efficiency is due to the more effective adjustment of the network’s weights and biases in the training phase. Conventional BP networks rely on gradient-based parameter updates, which may result in slow convergence and poor parameter tuning when there are nonlinear correlations. In contrast, the LM algorithm provides a better optimization approach for nonlinear least-squares problems, which allows more efficient updates of the network parameters. Therefore, the consistent improvement in all evaluation indices indicates that LM optimization enhances the BP network’s fitting accuracy and training stability.

The side-by-side placement of the five- and six-neuron configurations also illustrates the impact of hidden layer complexity on predictive power. For the LM-optimized network, increasing the hidden neurons from five to six decreased the maximum relative error from 0.98% to 0.71%, which resulted in the smallest total prediction errors. This suggests that for the dataset used in this study, the five-neuron configuration would not have sufficient nonlinear representational capacity to fully capture the relationships between pavement age, traffic load, climate factors and structural properties. Therefore, the six-neuron setup provides a better compromise between model complexity and nonlinear representative capabilities.

However, the six-neuron arrangement should be considered optimal only for the dataset and experimental parameters tested in this work. Increasing the number of hidden neurons indefinitely does not necessarily improve the predictive accuracy and can increase the computational costs and the risk of overfitting. Therefore, when the model is applied to pavement datasets with very different characteristics, the optimal hidden layer structure needs to be recalculated.

The enhancement of prediction accuracy is also very important from an engineering viewpoint because PQI is an inclusive measure of the effectiveness of pavement service. Improved PQI forecasting can provide road authorities with better insight into the anticipated deterioration of some pavement sections. This data can be used to identify roadway segments that need further investigation and to help prioritize preventive or condition-based maintenance. Consequently, the suggested model possesses the capability to function as a decision-support instrument for pavement management by delivering quantitative insights on pavement performance patterns and facilitating a more effective distribution of scarce maintenance resources.

The proposed model should be considered as an adjunct to on-site evaluation or engineering judgment rather than a substitute for it. Maintenance decisions are based on the structural integrity of the facility, safety regulations, financial constraints and regional maintenance criteria.

### 6.2. Future Research Directions

(1) Future research should increase the geographical coverage of the database to include asphalt pavement performance data from a larger range of regions and climate conditions. The external validation unrelated to the original study should use datasets from different countries, climatic regions and pavement infrastructures to fully evaluate the transferability and applicability of the models presented.
(2) Subsequent studies should consider other factors that may affect pavement deterioration, such as freeze–thaw cycles, characteristics of heavy vehicle traffic, asphalt binder properties, mixture gradation, drainage conditions, indicators of construction quality, timing of maintenance, and intensity of maintenance. These added variables may help to improve the detection of the pavement degradation mechanism-dependent and region-specific processes.
(3) Further studies are needed to extend the proposed models for pavement networks under different climatic conditions, materials, traffic characteristics and construction practices. If the new data are significantly different from the original training data, then the model may need to be recalibrated, retrained regionally or adapted to the new domain to maintain prediction accuracy. More reliable and practical prediction models of pavement performance can be achieved through the inclusion of systematic external validation and improved pavement performance metrics.
(4) Future research should aim at improving the pavement performance database by incorporating a broader spectrum of pavement condition states, particularly those representing moderate and poor conditions with significantly reduced PCI values. The inclusion of pavement sections in different stages of deterioration can provide a more comprehensive evaluation of the proposed models during the life cycle of the pavement. The predictive efficacy will be independently tested for different tiers of pavement condition to determine if the model has consistent accuracy with increased pavement deterioration. A better dataset will provide more convincing evidence of the generalization potential and practical application of the proposed models for pavement management systems.

### 6.3. Broad Applicability and Limitations

A BP neural network model and BP neural network models optimized using BP and LM were proposed, based on the asphalt pavement performance data from the LTPP database and pavement systems in multiple cities in China. Therefore, the applicability of the proposed models is mainly validated for pavement networks with climatic, traffic, structural, material, and construction features similar or closely correlated to those represented in the training dataset.

The developed models can be used for other pavement networks as long as the ranges and distributions of the seven input variables (pavement age, average annual daily traffic (AADT), annual temperature variation, annual rainfall, relative humidity, pavement thickness, and surface compressive strength) are sufficiently similar to those of the training data. However, when these variables are well out of the region of the training data, the model should be used with caution. Sometimes immediate deployment may decrease the accuracy of predictions, and the model may need to be recalibrated, retrained in the region or adapted for a new domain.

The applicability of the suggested models might be affected by climate, pavement material, traffic, structural design, construction method and maintenance practice. Therefore, the models might not be applicable in all cases. For example, pavement sections exposed to frequent freeze–thaw cycles or large temperature variations may experience deterioration processes not sufficiently represented in the existing training data. Similarly, variations in the asphalt binder properties, mixture gradation, pavement structural design, traffic composition, drainage conditions, and maintenance approaches can influence the relationship between the input variables and pavement deterioration. Therefore, the models proposed in this study should not be expected to provide consistent and reliable predictions for pavement networks with substantially different environmental or engineering conditions in the absence of independent validation.

Another important limitation is the representativeness of the pavement condition distribution in the existing dataset. The PCI values of the studied pavement segments are mostly high, often above about 75–80, indicating that the present dataset predominantly comprises pavements in satisfactory or somewhat satisfactory condition. Thus, the presented models have been mainly trained and tested on pavement segments with low to moderate degradation, while their predictive capability for pavements with medium or poor conditions is not sufficiently validated. Since pavement deterioration may exhibit different nonlinear characteristics at different stages of the pavement life cycle, the prediction efficiency demonstrated in this dataset cannot be assumed valid for pavement sections that are at much worse deterioration and have much lower PCI values. Therefore, the proposed models need to be used carefully on pavement sections with a condition that is far from the range of the training dataset.

Another limitation is the geographic coverage of the present dataset, which may not adequately represent the variety of pavement networks in different climates and engineering contexts. In addition, the seven input variables used in the final model include critical traffic, climatic, structural and aging factors, but some other factors with possible influence, such as the number of freeze–thaw cycles, composition of heavy vehicle traffic, properties of asphalt binder, mixture gradation, drainage conditions, quality of construction, time of maintenance and intensity of maintenance, were not explicitly included in the final model. Such constraints may limit the applicability of the proposed models to pavement networks that are significantly different.

In general, the proposed models should be considered as reliable mainly for the range of pavement conditions, input variable distributions, and environmental and engineering characteristics represented in the training dataset. Independent external evaluation should support wider implementation and regional recalibration or retraining, if needed, for pavement networks with different deteriorated conditions or significantly different climate, traffic, material, and construction factors.

## 7. Conclusions

The study developed an LM-optimized BP neural network architecture for predicting the service performance of asphalt pavements based on the PQI. The proposed framework includes seven quantitative determinants, including pavement age, AADT, annual temperature variation, yearly precipitation, relative humidity, pavement thickness and surface compressive strength. The main contribution of this work is the proposal of a nonlinear pavement performance prediction framework integrating traffic, climatic and structural data with LM-based optimization of the BP neural network, and a thorough study of the effect of the hidden-layer configuration and optimization strategy on prediction performance.

The comparison results show that LM optimization can effectively optimize the network weights and biases and improve the convergence property of the training process, so as to significantly improve the prediction performance of the traditional BP neural network. The LM-BP model with six hidden nodes achieved the best overall predictive accuracy among the studied configurations, with a maximum relative error of 0.71%. This result indicates that six hidden nodes are a good compromise between the network’s ability to capture nonlinearity and the complexity of the model for the dataset studied in this research. The optimal number of hidden nodes depends on the dataset and needs to be re-evaluated when the model is used on pavement datasets with much different characteristics.

The proposed model provides a feasible decision support approach for asphalt pavement performance management in practical application. Better PQI forecasting can support transportation agencies in pinpointing pavement segments that are vulnerable to rapid deterioration, enabling agencies to focus on detailed evaluations and to adopt proactive, condition-based maintenance approaches. With quantitative data on the expected performance of different pavement segments, the model could be used to improve the effective distribution of scarce maintenance resources. However, the model should be used as an additional decision-making support tool and should not replace field inspections, structural evaluations, or engineering judgment in actual pavement repair decisions. The proposed model is applicable depending on the characteristics and coverage of the training data. The model explicitly accounts for traffic and climatic factors, but the resulting framework is expected to perform best for pavement networks with traffic volumes, climatic conditions, pavement designs and material properties similar to or matching those represented in the training dataset. The prediction accuracy for pavement networks under very different environmental conditions, high traffic loads, construction techniques or paving materials has not been validated.

It is, therefore, necessary to recognize the many constraints of this study. The existing dataset is small in size and geographically limited, which constrains the model’s ability to generalize. Secondly, the nonlinear fitting accuracy of the BP neural network implies that the risk of overfitting cannot be completely ruled out from the current findings, however, the model has obtained high predictive accuracy. Third, other potentially impactful elements (e.g., freeze–thaw cycles, sun radiation, drainage conditions, specific material composition, axle load distributions, and maintenance histories) were not explicitly included in the final model. Future research should enlarge the database to include a broader spectrum of meteorological and traffic conditions, employ k-fold cross-validation with independent external validation, and incorporate further environmental, material and maintenance related variables. Sensitivity analysis and uncertainty quantification should be considered to improve the evaluation of robustness and transferability of the proposed model.

## Figures and Tables

**Table 1:** Original database variables and their roles.

Variable	Available in Original Database	Used in Final BP Model	Reason
Road ID	Yes	No	Identification only
Road length	Yes	No	Not selected as predictor
Construction date	Yes	Yes	Used to calculate road age
Maintenance records	Yes	No	Inconsistent classification/timing
Construction quality	Yes	No	Insufficient quantitative consistency
AADT	Yes	Yes	Traffic loading
Temperature	Yes	Yes	Used to derive annual temperature difference
Precipitation	Yes	Yes	Climate factor
Relative humidity	Yes	Yes	Climate factor
Pavement thickness	Yes	Yes	Structural factor
Surface compressive strength	Yes	Yes	Material factor
PCI	Yes	No	Supplementary performance indicator
RQI	Yes	No	Supplementary performance indicator
PQI	Yes	Yes	Model output

**Table 2:** Final input and output variables of the BP neural network model.

Variable	Symbol	Unit	Role
Road age	Age	year	Input
Average annual daily traffic	AADT	veh/day	Input
Annual temperature difference	ΔT	°C	Input
Annual precipitation	P	mm	Input
Relative humidity	RH	%	Input
Pavement thickness	H	mm	Input
Surface compressive strength	CS	MPa	Input
Pavement Quality Index	PQI	-	Output

**Table 3:** Data on road performance indicators for selected pavements.

Section ID	Year	PQI	RQI	PCI
1	2019	89.82	89.18	91.34
	2020	86.18	85.61	90.57
	2021	84.37	83.88	89.92
	2022	83.85	82.37	87.06
	2023	82.94	81.24	85.63
2	2019	92.63	92.78	93.65
	2020	91.89	91.36	91.42
	2021	89.25	88.84	90.85
	2022	85.24	85.68	88.82
	2023	83.89	83.72	86.41
3	2019	88.67	88.35	89.27
	2020	87.75	87.60	87.84
	2021	84.53	84.28	84.92
	2022	81.38	81.27	81.19
	2023	79.83	79.62	79.97
4	2019	88.58	88.14	89.71
	2020	86.82	86.32	87.64
	2021	84.27	83.17	84.61
	2022	80.92	80.69	81.35
	2023	79.07	78.46	79.81
5	2019	87.27	86.54	87.62
	2020	84.29	84.37	85.14
	2021	81.72	81.25	82.01
	2022	79.84	79.15	80.27
	2023	76.72	77.16	78.32
6	2019	93.18	93.06	93.84
	2020	92.08	92.59	92.15
	2021	90.52	90.52	91.17
	2022	86.07	86.43	87.72
	2023	82.48	82.75	82.49

**Table 4:** Pavement thickness on selected road sections.

Section ID	Pavement Thickness (cm)
1	55
2	59
3	47
4	44
5	50
6	52

**Table 5:** Compressive strength values of the surface course on selected road sections.

Section ID	Surface Rebound Modulus of Road Surface (MPa)
1	1293.37
2	1392.23
3	1290.98
4	1210.87
5	1302.12
6	1258.74

**Table 6:** Summary of predicted values and relative errors before and after optimization.

Sample No.	Actual Value	Predicted Value of BP Network (5 Hidden Nodes)	Relative Error (RE, %)	Predicted Value of BP Network (6 Hidden Nodes)	RE (%)	Predicted Value of Optimized BP Network (5 Hidden Nodes)	RE (%)	Predicted Value of Optimized BP Network (6 Hidden Nodes)	RE (%)
1	89.82	91.48	1.85	90.63	0.90	90.38	0.62	90.17	0.39
2	86.18	84.39	2.08	87.12	1.09	85.46	0.84	86.61	0.50
3	84.37	83.02	1.60	85.63	1.49	84.88	0.61	84.67	0.36
4	83.85	85.21	1.62	83.11	0.88	83.41	0.52	84.09	0.29
5	82.94	81.35	1.92	83.95	1.22	82.21	0.88	83.39	0.54
6	92.63	94.21	1.71	93.41	0.84	93.33	0.76	92.89	0.28
7	91.89	90.25	1.78	92.78	0.97	91.08	0.88	92.18	0.32
8	89.25	87.96	1.45	88.65	0.67	89.97	0.81	89.48	0.26
9	85.24	86.49	1.47	84.22	1.20	84.55	0.81	85.78	0.63
10	83.89	82.38	1.80	84.56	0.80	84.61	0.86	83.56	0.39
11	88.67	90.12	1.64	87.25	1.60	89.01	0.38	88.12	0.62
12	87.75	86.21	1.75	88.56	0.92	86.98	0.88	88.11	0.41
13	84.53	83.18	1.60	85.19	0.78	85.30	0.91	84.89	0.43
14	81.38	79.25	2.62	82.24	1.06	80.91	0.58	81.96	0.71
15	79.83	78.26	1.97	80.35	0.65	80.55	0.90	79.42	0.51
16	88.58	90.25	1.94	87.63	1.30	89.36	0.88	88.07	0.58
17	86.82	85.14	1.61	87.95	0.75	86.22	0.69	87.39	0.66
18	84.27	82.91	2.22	83.64	1.52	83.63	0.76	84.70	0.51
19	80.92	79.12	2.01	82.15	1.04	81.71	0.98	80.46	0.57
20	79.07	77.48	2.04	78.25	1.17	78.43	0.81	79.54	0.59
21	87.27	89.05	2.53	86.25	1.27	87.86	0.68	87.58	0.36
22	84.29	82.16	1.73	85.36	0.77	84.71	0.50	84.81	0.62
23	81.72	83.13	1.52	81.09	0.85	81.17	0.67	81.97	0.31
24	79.84	78.63	2.66	80.52	1.19	79.12	0.90	80.09	0.31
25	76.72	74.68	1.49	77.63	1.04	77.45	0.95	76.98	0.34
26	93.18	94.57	1.77	92.21	1.09	93.72	0.58	92.79	0.42
27	92.08	90.45	0.93	93.08	1.52	92.73	0.71	91.58	0.54
28	90.52	91.36	1.99	89.14	1.77	91.32	0.88	90.21	0.34
29	86.07	84.36	1.36	87.59	0.81	86.76	0.80	85.58	0.57
30	82.48	81.36	1.39	83.15	1.23	81.89	0.72	82.93	0.55

## Data Availability

The data presented in this study are openly available in github at https://github.com/users/XIXIGUAINE/projects/5 (accessed on 1 July 2026), reference number [36].
